# Supplementary material for: Controllable synthesis of pomelo peel-based aerogel and its application in adsorption of oil/organic pollutants
Source: R Soc Open Sci. 2019 Feb 6;6(2):181823. doi: 10.1098/rsos.181823 (PMC6408386; doi:10.1098/rsos.181823)
Supplement: Support information for rsos [file rsos181823supp1.docx]

**Supporting Information**

Controllable synthesis of pomelo peel-based aerogel and its application in adsorption of oil/organic pollutants


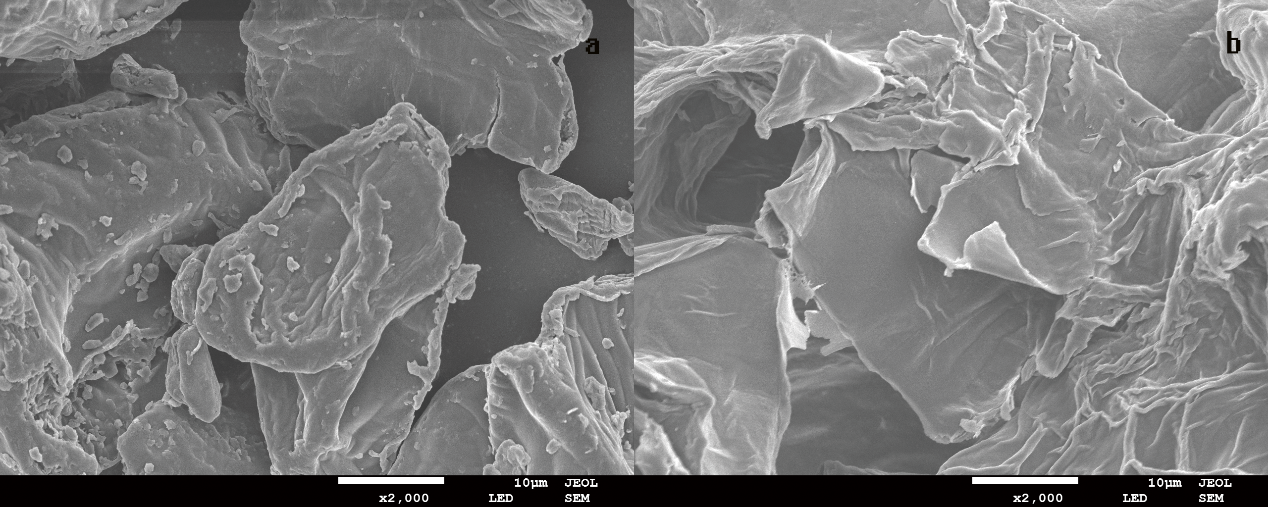


Figure S1. SEM images of PP and alkali pretreatment PP.


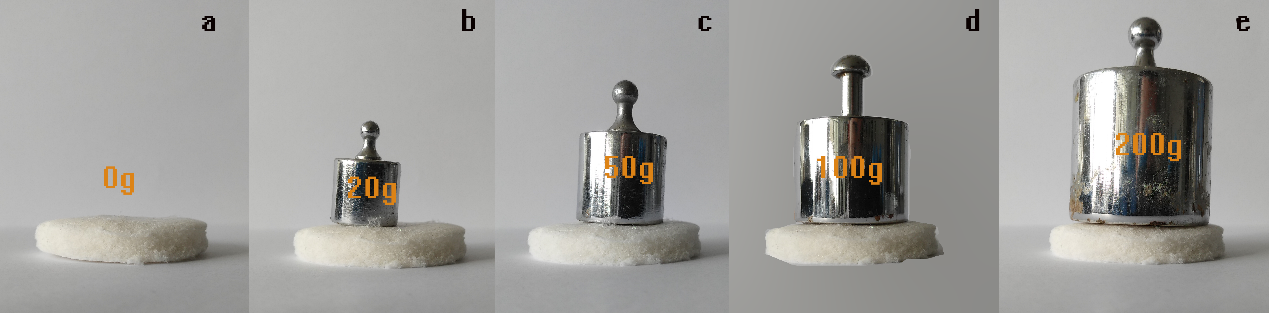


Figure S2. The mechanical strength of HPSA-1.


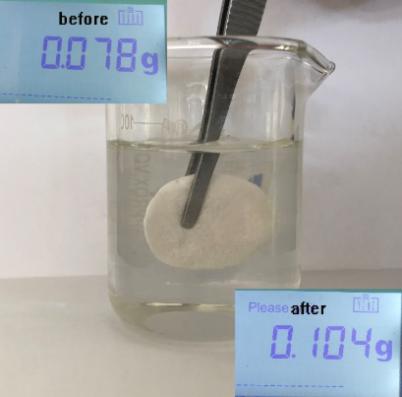


Figure S3. The image of sponge aerogel absorbing water.

As shown in Figure S3, the modified aerogels can absorb little water (the water absorption capacity was 0.33g/g) when we put the aerogel into water completely for 20S. It was due to the porous structure of aerogel, especially a large number of macrospores were distributed in the aerogel. Water can enter these macrospores and lead to residue by strong pressure force.


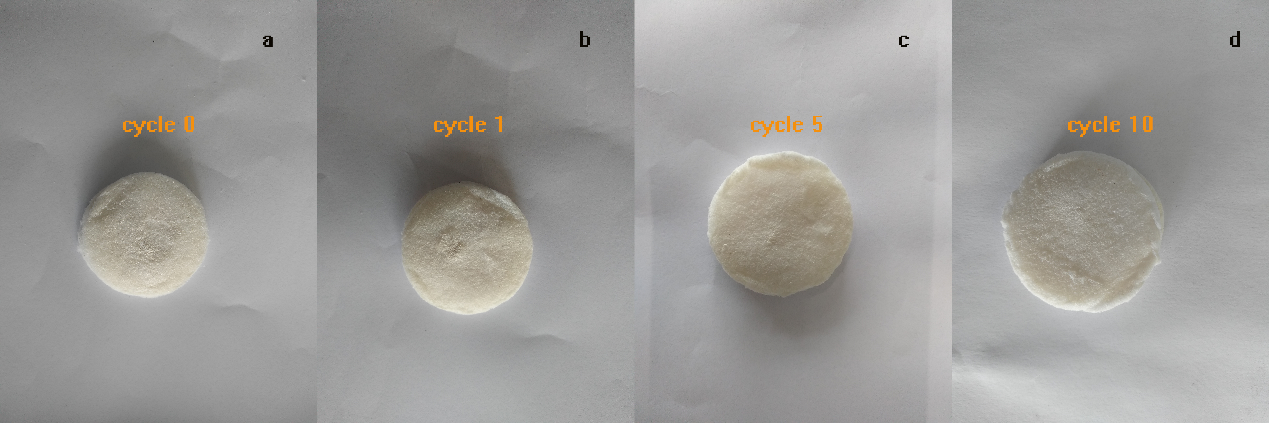


Figure S4. The Surface image of sponge aerogel for 10 cycles.





Figure.S5 The FTIR of a natural pomelo peel (PP).

As shown in Figure.S5, this is the FTIR spectrum of a natural pomelo peel. The bands in the region of 3400 cm^-1^ indicate the presence of a stretching of strong hydroxyl groups. The band at 2905 cm^-1^ is assigned to C-H stretching. The band at 1630 cm^-1^ represents absorbed water and the band at 1391 cm^-1^ assigned to -C-H. In addition, the band at 1061 cm^-1^ corresponds to C-OR stretching.
